# Outcomes after transabdominal cerclage in twin pregnancy with previous unsuccessful transvaginal cerclage

**DOI:** 10.1371/journal.pone.0232463

**Published:** 2020-04-30

**Authors:** Ga-Hyun Son, Heejin Ham, Sung-Taek Park, So-Yeon Choi, Ji-Eun Song, Keun-Young Lee

**Affiliations:** Division of Maternal-Fetal Medicine, Department of Obstetrics and Gynecology, Kangnam Sacred Heart Hospital, Hallym University College of Medicine, Seoul, Korea; University of Mississippi Medical Center, UNITED STATES

## Abstract

Transabdominal cerclage (TAC) is reported to be effective for preventing preterm birth in women with unsuccessful transvaginal cerclage (TVC) history. However, TAC has rarely been performed in twin pregnancy given the lack of sufficient evidence and the technical difficulty of the operation. Thus, it is unclear whether TAC is an effective procedure for twin pregnancy in women with a history of unsuccessful TVC. The aim of this study is to compare the characteristics and pregnancy outcomes after TAC in twin pregnancy versus singleton pregnancy, to examine whether twin pregnancy is a risk factor for very preterm birth (before 32 weeks) after TAC, and to determine whether TAC is effective in preventing preterm birth in twin pregnancy. This single-center retrospective cohort study included women who underwent TAC because of unsuccessful TVC history between January 2007 and June 2018. Of 165 women who underwent TAC, 19 had twins and 146 had singletons. Our results showed that the neonatal survival rate improved dramatically when TAC was performed (15.4% (prior pregnancy) vs 94.0% (after TAC) in twins, p<0.01; 22.8% (prior pregnancy) vs 91.1% (after TAC) in singletons, p<0.01). Moreover, the risk of very preterm birth was significantly decreased after TAC in both groups (36/39 (92.3%) (prior pregnancy) vs 2/19 (10.5%) (after TAC) in twins, p<0.01; 290/337 (86.1%) (prior pregnancy) vs 17/146 (11.6%) (after TAC) in singletons, p<0.01). More advanced maternal age and history of prior preterm delivery between 26+0 and 36+6 weeks were independently associated with very preterm birth, whereas the presence of a twin pregnancy was not associated with very preterm birth on multivariate logistic regression analysis. These results suggest that TAC is associated with successful prevention of very preterm birth and improved neonatal survival rates in the absence of procedure-related major complications in women with twin pregnancy and previous unsuccessful TVC history.

## Introduction

The incidence of twin pregnancy continues to increase, and now accounts for more than 3% of all live births [1]. The most serious risk in twin pregnancy is preterm delivery, which plays a major role in the increased perinatal mortality and morbidity. Almost 60% of twins are born prematurely, and 11% of twins are delivered before 32 weeks compared to 1.6% of singletons [2]. One factor leading to preterm delivery is cervical insufficiency, which typically presents as painless cervical dilatation in the mid-trimester. It occurs in about 0.5%-1% of all pregnancies but accounts for 10%-25% of second-trimester pregnancy losses [3–5]. An extreme manifestation of cervical insufficiency occurs in women with multiple prior second-trimester losses or preterm births who received a history-indicated cerclage, but who delivered before 33 weeks despite this intervention, i.e., unsuccessful transvaginal cerclage (TVC) [6]. Successful outcomes of transabdominal cerclage (TAC) have been reported in women with singleton pregnancy and a history of unsuccessful TVC or with a traumatized cervix that precludes TVC [6–10]. However, given the increased procedure-related morbidity and the need for two laparotomies, most experts strictly confine the indication of TAC to women who run out of options for continuing the pregnancy, especially with twin pregnancy [8]. Although the incidence of cervical insufficiency in multiple gestations was reported significantly higher than that among women with singleton pregnancy [5,11–14], the management of women with twin pregnancy with cervical insufficiency has not been established. Moreover, the management of women with twin pregnancy who have a history of unsuccessful TVC is challenging. Only a few case reports have examined the use of TAC in twin pregnancy [8,15–22]. Therefore, given the lack of sufficient evidence, it is unclear whether TAC is an effective procedure in women with twin pregnancy and unsuccessful TVC history, and the ability to counsel such women is limited. Thus, the aim of this study is to compare the characteristics and pregnancy outcomes after TAC in twin pregnancy with those of singleton pregnancy, to examine whether twin pregnancy is a risk factor for very preterm birth (before 32 weeks) after TAC, and to determine whether TAC is effective in preventing preterm birth in twin pregnancy.

## Materials and methods

**Fig 1:**
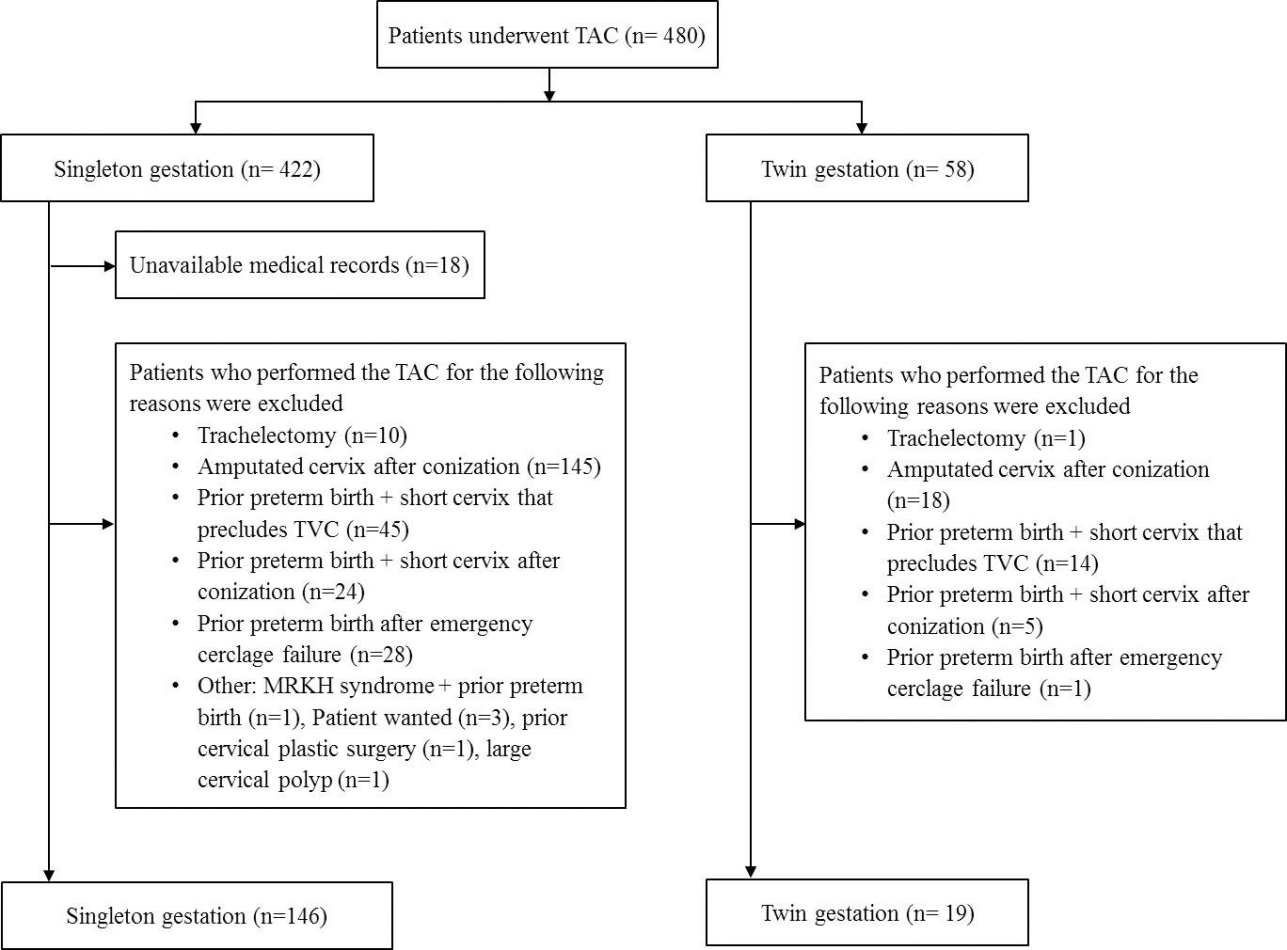
Detailed reasons of exclusion and number of patients who underwent transabdominal cerclage. TAC, transabdominal cerclage; TVC, transvaginal cerclage; MRKH, Mayer-Rokitansky-Küster-Hauser.

A total of 480 patients were identified through a search of medical records for TAC procedure between January 2007 and June 2018 at Hallym University Medical Center. The study population included women with unsuccessful TVC history, and women with TAC indicated for an extremely short or severely deformed cervix by extensive conization or trachelectomy were excluded from this study. Women were also excluded if cerclage was placed before the pregnancy. Previous unsuccessful TVC was defined as a preterm birth before 33 gestational weeks despite history-indicated cerclage in the previous pregnancies. Detailed reasons of exclusion and number of patients are presented in Fig 1. Finally, 165 patients who underwent TAC for prior unsuccessful TVC were analyzed.

The medical records of all patients were reviewed and data regarding TAC and delivery were abstracted. TAC was performed between the end of the first trimester and early period of the second trimester of pregnancy (between 12 and 17 gestational weeks). Gestational age, detectable major fetal anomalies, cervix status, and chorionicity and discordancy of fetal size in twin pregnancy were assessed by ultrasonography. Chorionicity was confirmed by postnatal placental histology. Before surgery, none of the patients had experienced abnormal uterine contraction, rupture of membranes, or vaginal bleeding. Outcome data included complications of surgery, gestational age at delivery, and neonatal outcomes. All women were counseled sufficiently before the procedure regarding the potential risks and benefits of TAC. Institutional Review Board approval for the collection of data was obtained from Hallym University Medical Center (No. 2014-04-52); the need for informed consent was waived due to the retrospective design.

### Transabdominal cerclage procedure

All TAC operations were performed by one surgeon (L.K.Y) using the technique of Benson and Durfee with modifications, as described previously [7,8,23]. All procedures were performed under general anesthesia through a Pfannenstiel incision. Cephalosporin was given intravenously before incision. The vesicouterine peritoneum was incised and the bladder was pushed downward to expose the cervicoisthmic junction. The uterus was exteriorized, and the avascular area between the ascending and descending branches of the uterine artery and isthmus was identified by palpation, and a puncture was made with a right-angle clamp entering from anterior to posterior, medial and superior to the uterosacral ligaments. A 5-mm Mersilene tape was grasped with the open right-angle clamp and passed from posterior to anterior. The procedure was then repeated on the other side. The suture was pulled tight and tied anteriorly. The puncture sites were sutured with a figure-8 of 1–0 Vicryl for hemostasis. After hemostasis was confirmed, the uterus was returned to the pelvic cavity, and the peritoneal bladder fold was closed over the suture. We performed an elective cesarean section at 36–38 weeks gestation in twins and at more than 37 weeks in singleton. Patients were given the option of leaving the cerclage suture in place or removing the suture before the operation.

### Statistical analysis

Data are shown as median (interquartile range; IQR) or mean ± standard deviation (SD) for continuous variables, and as n (%) for categorical variables. Comparisons were made between twin and singleton pregnancies using Mann-Whitney U-test or Student’s t-test for continuous variables, and Fisher’s exact test, or χ^2^ tests for categorical variables. Patient characteristics associated with very preterm birth (< 32 weeks) and late preterm birth (< 36 weeks) were identified in bivariable analysis. The presence of a twin pregnancy was forced into the model, and a multivariable logistic regression was then performed to identify risk factors associated with very preterm birth and late preterm birth, and to determine that twin pregnancy is an independent risk factor for preterm birth after TAC. Statistical analysis was conducted using SPSS software (version 23.0, IBM, Armonk, NY, USA). A P value of < .05 was considered statistically significant.

## Results

During the study period, 480 women underwent TAC placement: 58 (12.1%) had twins and 422 (87.9%) had singletons. Of the 480 identified women, 165 women who underwent TAC because of unsuccessful TVC history were included in this study. Of the 165 women, 19 had twins and 146 had singletons, and all cases of twin pregnancy were dichorionic diamniotic twin. The obstetric history of all patients selected for TAC is presented in Table 1, and detailed clinical data of twin pregnancy are summarized in Table 2. In this study, no differences in gestational age at TAC placement, neonatal survival rate in prior pregnancies, and prior pregnancy losses before 16 weeks were found between the two groups. Prior preterm delivery during the early second trimester (16+0 to 25+6 weeks) in both twins and singletons were more common than in the late second and third trimesters (26+0 to 36+6 weeks). Most of the patients had undergone one or more attempts at TVC including history-indicated cerclage in previous pregnancies, and the most frequent cases of attempted cerclage were four times in singleton and three times in twins, and 61 women with singletons and seven with twins had two or more cases of attempted cerclage. Mullerian anomaly in women with singletons included uterine didelphys in three women and bicornuate uterus in four women.

**Table 1:** Patient characteristics selected for transabdominal cerclage.

	Twin (n = 19)	Singleton (n = 146)	*P* value
Maternal age (years)	32.0 (30.0–35.0)	33.0 (31.0–36.0)	0.29
Gestational age at TAC (weeks)	13.6 (13.3–14.1)	13.6 (13.2–14.3)	0.68
Hypertension, n (%)	0	3 (2.1)	1.00
Diabetes Mellitus	2	7 (4.8)	1.00
Previous pregnancies			
Number of total pregnancies	52	425	
Pregnancy losses before 16 weeks	13	88	
Preterm delivery			
16+0 to 25+6 weeks	29	262	
26+0 to 36+6 weeks	7	48	
Gestational age at preterm delivery (weeks)	21.0 (18.6–23.4)	21.0 (19.0–24.0)	0.79
Fullterm delivery (≥ 37+0 weeks)	3	27	
Total neonatal survival*	6/39 (15.4)	77/338 (22.8)	0.32
Previous failed TVC			
History-indicated cerclage	19	162	
Total number of cerclages	27	216	
Gestational age at TVC (weeks)	14.0 (13.8–17.7)	14.0 (13.0–16.0)	0.22
Previous TAC	0	2	
Mullerian anomaly	0	7 (4.8)	1.00
Prior cervical surgery, LEEP/CKC, n (%)	0	5 (1.8)	1.00

TAC. transabdominal cerclage; LEEP/CKC, loop electrosurgical excisional procedure/cold knife cone. Data are presented as n, n (%), or median (interquartile range)

* Total neonatal survival, pregnancy losses before 16 weeks excluded.

**Table 2:** Obstetric history of women with twin pregnancy selected for transabdominal cerclage.

No.	First trimester loss	Second trimester loss <26 weeks	Spontaneous preterm delivery 26–36 weeks	≥37 weeks	Surviving children	Failed previous TVC
1	1	2 (21w, 23w)	0	0	0	1 (H)
2	1	2 (22w, 23w)	0	0	0	1 (H)
3	2	1 (25w)	0	0	0	2 (H + U)
4	0	2 (18w, 19w)	0	0	0	1 (H)
5	0	2 (20w, 23w)	0	1 (neonatal death)	0	1 (H)
6	0	1 (19w)	1 (26w)	0	0	1 (H)
7	0	2 (19w, 21w)	0	0	0	1 (H)
8	1	2 (16w, 22w)	0	0	0	2 (H + P)
9	2	0	1 (27w, neonatal death)	1	0	1 (H)
10	1	2 (17w, 20w)	0	0	0	2 (H + P)
11	0	2 (18w, 21w)	1 (27w, fetal death)	0	0	2 (H + U)
12	0	1 (19w)	1 (26w, neonatal death)	0	0	2 (H + P)
13	3	2 (18w, 19w)	0	0	0	1 (H)
14	0	1 (17w)	1 (27w)	0	1	1 (H)
15	0	1 (16w)	1 (26w)	1	2	1 (H)
16	0	1 (20w)	1 (28w)	0	1	2 (H + P)
17	0	2 (18w, 23w)	0	0	0	1 (H)
18	0	2 (20w, 23w)	0	0	1	1 (H)
19	2	1 (25w)	0	0	0	3 (H+ P 2 times)

TVC, Transvaginal cerclage; w, weeks; H, history-indicated cerclage; U, Ultrasound-indicated cerclage; P, Physical examination-indicated cerclage.

Pregnancy outcomes following TAC are presented in Table 3. Gestational age at delivery differed between the two groups, with twins delivering at a median 35.4 weeks (IQR, 34.4–36.4) compared with singletons at 38.1 weeks (IQR, 37.4–38.3) (P<0.01). Rates of delivery before 24 and 32 weeks did not differ. Thus, this difference was primarily related to differences in delivery at gestational ages later than 32 weeks. Women with twins delivered more likely between 32 and 35 6/7 gestational weeks, and women with singletons delivered more likely later than 36 gestational weeks. Three women with twin pregnancy delivered during 32–35 6/7 gestational weeks because of iatrogenic causes (diagnoses of preeclampsia (n = 2), oligohydramnios, and discordancy of fetal weight (n = 1)), and five women delivered preterm because of preterm labor (n = 4) and preterm premature rupture of membranes (PPROM) (n = 1). Both iatrogenic and spontaneous preterm birth between 32 and 35 6/7 gestational weeks were more common in twin pregnancy than in singleton pregnancy. Neonatal birth weight was less in twin neonates compared with their singleton counterparts. Rates of live birth, neonatal survival, and PPROM did not differ between the two groups. In both groups, the neonatal survival rate has improved dramatically when TAC was performed (15.4% (prior pregnancy) vs 94.0% (after TAC) in twins, p<0.01; 22.8% (prior pregnancy) vs 91.1% (after TAC) in singletons, p<0.01). Moreover, the risk of very preterm birth was significantly decreased after TAC in both groups (36/39 (92.3%) (prior pregnancy) vs 2/19 (10.5%) (after TAC) in twins, p<0.01; 290/337 (86.1%) (prior pregnancy) vs 17/146 (11.6%) (after TAC) in singletons, p<0.01).

**Table 3:** Pregnancy outcomes of transabdominal cerclage.

	Twin (n = 19)	Singleton (n = 146)	*P* value
Gestational age at delivery	35.4 (34.4–36.4)	38.1 (37.4–38.3)	<0.01
< 24 weeks	1 (5.3)	13 (8.9)	1.00
24–31 6/7 weeks	1 (5.3)	4 (2.7)	0.46
32–35 6/7 weeks	8 (42.1)	6 (4.1)	<0.01
Iatrogenic	3 (15.8)	3 (0.2)	0.02
Spontaneous	5 (26.3)	3 (2.1)	<0.01
≥36 weeks	9 (48.0)	123 (84.2)	<0.01
Live birth	36/38 (94.7)	134 (91.8)	0.74
Neonatal survival	35/38 (94.0)	133 (91.1)	1.00
Birth weight	2.3 (2.0–2.6)	3.2 (2.9–3.5)	<0.01
	2.3 (1.7–2.7)		<0.01
PPROM	3 (15.8)	12 (8.2)	0.39

PPROM, preterm premature rupture of membranes.

Data are presented as median (interquartile range) or n (%).

Next, we examined the patient characteristics associated with preterm birth after TAC. We divided preterm delivery into very preterm and late preterm, then bivariable analysis was performed for each. More advanced maternal age and history of prior preterm delivery between 26+0 and 36+6 weeks were associated with increased odds of very preterm birth (odds ratio [OR], 1.15; 95% confidence interval [CI], 1.02–1.30, OR, 3.13; 95% CI, 1.18–8.28, respectively). On the other hand, more advanced maternal age and twin pregnancy were associated with increased odds of spontaneous late preterm birth (OR, 1.13; 95% CI, 1.01–1.26, OR, 4.73; 95% CI, 1.65–13.59, respectively). We forced the presence of twin pregnancy into the logistic regression model as a factor along with other significant risk factors for preterm birth, and the results for the multivariable regression analysis are shown in Table 4. More advanced maternal age and history of prior preterm delivery between 26+0 and 36+6 weeks were independently associated with very preterm birth, but the presence of a twin pregnancy was not associated with very preterm birth. However, the presence of twin pregnancy and more advanced maternal age were independently associated with spontaneous late preterm birth. Thus, twin pregnancy may be a risk factor for spontaneous late preterm birth after TAC but not associated with very preterm birth.

**Table 4:** Multivariable analyses of factors associated with preterm birth.

Variable	Preterm birth < 32 weeks			Preterm birth < 36 weeks		
	aOR	95% CI	*P* value	aOR	95% CI	*P* value
Maternal age	1.16	1.01–1.32	0.04	1.17	1.03–1.33	0.02
History of prior preterm delivery between 26+0 and 36+6 weeks	3.07	1.12–8.44	0.03	2.11	0.88–5.08	0.10
Twin pregnancy	0.92	0.19–4.51	0.92	5.99	1.98–18.1	< 0.01

aOR, adjusted odds ratio; CI, confidence interval; GA, gestational age.

Complications of TAC such as intraoperative hemorrhage, intrauterine fetal death, and rupture of membranes within 1 week of the surgery in twins were similar to those in singletons (Table 5). The average operation time and hospital day (55.5±11.5 (twins) vs 52.7±12.5 (singleton), 7.7±2.0 (twins) vs 7.6±1.2 (singleton), respectively) did not differ between the two groups. Intraoperative hemorrhage >500 mL occurred in one patient in the twin group and in 5 women in the singleton group. Severe intraoperative hemorrhage was caused by rupture of parametrial veins; in every case, the bleeding was stopped after tying off the band, followed by some hemostatic sutures. One woman with a singleton pregnancy and 800 mL of intraoperative hemorrhage had a history of three unsuccessful history-indicated cerclages, one emergency cerclage, and failure of laparoscopic cervicoisthmic cerclage; consequently, she had PPROM 2 weeks following TAC placement, and resulting in pregnancy loss at 15+6 weeks. Except for this patient, the remaining women with severe intraoperative hemorrhage delivered at >34 gestational weeks. In pregnancies that were difficult to maintain until gestational age of fetal viability because of PPROM, intrauterine fetal death, or uncontrolled preterm labor, we performed dilatation and evacuation before 16 gestational weeks (n = 7); thereafter, we performed hysterotomy (n = 5) in singleton pregnancy, and we performed hysterotomy in a woman with a twin pregnancy at 14 gestational weeks due to PPROM 1 week following TAC. All operations were done without major complications such as serious maternal morbidity and mortality. The cerclage suture was removed during cesarean section in 66 women (45.2%) with singleton and in 13 women (68.4%) with twin (p = 0.06) pregnancies as per patient’s request, with no complications encountered during the procedure. No long-term complications, such as cervical erosion, were reported in any of the remaining patients with retained cerclage suture.

**Table 5:** Complications of transabdominal cerclage.

	Twin (n = 19)	Singleton (n = 146)	*P* value
Complications of transabdominal cerclage			
Blood loss (mL)	135.8±133.9	128.5±109.9	0.79
Blood loss ≥ 500 mL	1 (5.3)	5 (3.4)	0.53
Operation time (min)	55.5±11.5	52.7±12.5	0.32
Intrauterine fetal death	0	4 (2.7)	1.00
Rupture of membranes within one week of the surgery	1 (5.3)	5 (3.4)	0.53
Hospital day (day)	7.7±2.0	7.6±1.2	0.72

Data are presented as mean±standard deviation or n (%).

## Discussion

In this study, we have identified 19 women with twin pregnancy who underwent TAC for previous unsuccessful TVC and documented the characteristics of prior pregnancies as well as their pregnancy and operative outcomes following TAC. Moreover, we have analyzed these factors compared to 146 singleton pregnant women who underwent TAC during the same period, and identified that women with twin pregnancy who received TAC have comparable characteristics and obstetric histories with their singleton counterparts. In addition, there were no significant differences in perioperative management such as tocolytics, antibiotic use, and surgical technique between two groups.

TAC is indicated for patients in whom cerclage, although required, cannot be placed because of anatomical limitations of the cervix or in cases of prior unsuccessful TVC procedures [6–10]. Although no appropriate control patients have been included in previous studies, 21 separate studies examining the use of TAC have been reported, almost all with excellent results, with an overall success rate of 89% [6–10,24]. In patients with unsuccessful TVC history, TAC was associated with significantly lower incidences of preterm delivery and PPROM compared with patients who underwent TVC [6]. However, despite its relative success in singleton pregnancy, TAC is rarely performed in twin pregnancy. A systemic review of the literature in PubMed and EMBASE for the terms “twin pregnancy” and “transabdominal cerclage” revealed eight publications presenting a total of 16 cases of twin pregnancy [15–20,22]. These cases resulted in 28/32 (87.5%) successful pregnancies and 4/32 (12.5%) perinatal deaths. The overall results from this study were favorable and similar to previous studies. Following TAC, the neonatal survival rates have improved dramatically and the risk of very preterm birth was greatly reduced in both singleton and twin groups. 89.5% of women in the twins and 88.4% in the singletons gave birth after 32 gestational weeks following TAC, and the median birth weight was >2.0 kg in both groups. Although twins were delivered earlier than singletons, considering that the incidence of very preterm delivery for twin is 11% and the median duration of pregnancy is 35.4 weeks (IQR, 34.4–36.4) for twins, our pregnancy outcomes are comparable to the general twin pregnancy outcomes [25]. Moreover, our results showed that the rates of very preterm birth did not differ between the two groups and the presence of twin pregnancy was not associated with very preterm birth after TAC on multivariable logistic regression analysis. Thus, we can corroborate that TAC is effective in preventing very preterm birth in twin pregnancy with unsuccessful TVC history. Despite the apparent efficacy of TAC, it is a technically demanding procedure, particularly in twins, with significant risk of morbidity as it requires two laparotomies. Furthermore, twin pregnancy has more uterine expansion, so it is more difficult to externalize the uterus during surgery, more bleeding may occur, and there could be a greater chance of irregular contractions or rupture of membranes after surgery. Thus, all patients considering TAC should be made aware of the risks and provided with sufficient counseling to help weigh the potential positive and negative outcomes associated with this procedure.

In our study, intrauterine fetal deaths occurred in four women with singletons, not with twins. Although no particular complications during operation were recorded in all cases of intrauterine fetal death, one case of fetal death following membrane rupture occurred 1 day after operation at 17 gestational weeks, and we are not sure whether the late operation was related with the fetal death. Late prenatal complications including suture migration, uterine rupture, rectovaginal fistula, and persistent maternal discomfort have not been reported. Although bleeding from parametrial veins is the main perioperative complication in reported studies, in our experience, the vessel-free area can be accurately determined by palpation [26–28]. Similarly, during puncture of the paracervical tissue, the uterine vessels and ureters can be avoided using a gentle lateral force. Thus, major bleeding did not occur in most patients, and in those where bleeding did occur, it was readily controlled by tying off the band and applying hemostatic sutures.

This study includes data of only women who had experienced TVC failure and excludes women with short or traumatized cervix that precludes TVC; We can analyze pregnancy outcomes after TAC in twin pregnancy more accurately by excluding patients whose cervical factors were indications for TAC, because cervical factors such as traumatized, short, amputated, or marked scarring and defect vary widely in form, cause, and degree, and each of them has different effects on pregnancy outcomes. Moreover, although the study population has homogenous indications for TAC, a relatively large number of patients were included. These enabled us to compare pregnancy outcomes and perioperative complications between twins and singletons and to analyze the efficacy of TAC in twins.

This study has limitations. First, since this study spanned a relatively long period, there may be a difference in pregnancy outcomes according to the period. However, it is unlikely that the time frame would bias the data because the improvements over time in prolongation of pregnancy after TAC or risk of preterm birth have not been recognized, and there have been little change in perioperative management strategies, such as the use of antibiotics or tocolytics. Second, TAC is a procedure that requires rich experience and surgical skills particularly in twins and pregnancy outcome can vary depending on surgeon’s skill. All TAC was performed by a single obstetrical surgeon in our study. Thus, there could be limitations to generalizing our results in other groups. In addition, previous failed TVC was performed in 24 (14.5%) patients at our hospital. Thus, in many cases, prior TVC were performed in other hospitals, and accordingly, information on previous pregnancies provided by patients could be inaccurate, which may have influenced the results. Lastly, our study does not evaluate the best candidate for TAC in twins. We performed TAC for various indications. Although excluded from the analysis, we have performed TAC in women with twin pregnancy, prior preterm birth, and short cervix that precludes TVC (n = 19, Fig 1). However, it is unclear whether this is beneficial to extend pregnancy compared to expectant management.

## Conclusion

In women with twin pregnancy with previous unsuccessful history-indicated cerclage history, TAC appeared not only to significantly increase the neonatal survival rate, but also to be effective in preventing very preterm birth. The results of this study may be helpful in counseling the woman with a twin pregnancy who is suspected of cervical insufficiency with previous unsuccessful TVC history regarding her treatment options.

## Supporting information

S1 Raw Data (XLSX)
